# Rescue of placental phenotype in a mechanistic model of Beckwith-Wiedemann syndrome

**DOI:** 10.1186/1471-213X-10-50

**Published:** 2010-05-11

**Authors:** Rosemary Oh-McGinnis, Aaron B Bogutz, Kang Yun Lee, Michael J Higgins, Louis Lefebvre

**Affiliations:** 1 Department of Medical Genetics, Molecular Epigenetics Group, Life Sciences Institute, University of British Columbia, Vancouver, Canada; 2 Department of Molecular and Cellular Biology, Roswell Park Cancer Institute, Buffalo, USA

## Abstract

**Background:**

Several imprinted genes have been implicated in the process of placentation. The distal region of mouse chromosome 7 (Chr 7) contains at least ten imprinted genes, several of which are expressed from the maternal homologue in the placenta. The corresponding paternal alleles of these genes are silenced in *cis *by an incompletely understood mechanism involving the formation of a repressive nuclear compartment mediated by the long non-coding RNA *Kcnq1ot1 *initiated from imprinting centre 2 (IC2). However, it is unknown whether some maternally expressed genes are silenced on the paternal homologue via a *Kcnq1ot1-*independent mechanism. We have previously reported that maternal inheritance of a large truncation of Chr7 encompassing the entire IC2-regulated domain (DelTel7 allele) leads to embryonic lethality at mid-gestation accompanied by severe placental abnormalities. *Kcnq1ot1 *expression can be abolished on the paternal chromosome by deleting IC2 (IC2KO allele). When the IC2KO mutation is paternally inherited, epigenetic silencing is lost in the region and the DelTel7 lethality is rescued in compound heterozygotes, leading to viable DelTel7/IC2KO mice.

**Results:**

Considering the important functions of several IC2-regulated genes in placentation, we set out to determine whether these DelTel7/IC2KO rescued conceptuses develop normal placentae. We report no abnormalities with respect to the architecture and vasculature of the DelTel7/IC2KO rescued placentae. Imprinted expression of several of the IC2-regulated genes critical to placentation is also faithfully recapitulated in DelTel7/IC2KO placentae.

**Conclusion:**

Taken together, our results demonstrate that all the distal chromosome 7 imprinted genes implicated in placental function are silenced by IC2 and *Kcnq1ot1 *on the paternal allele. Furthermore, our results demonstrate that the methylated maternal IC2 is not required for the regulation of nearby genes. The results show the potential for fully rescuing *trans *placental abnormalities that are caused by imprinting defects.

## Background

Genomic imprinting is the mechanism by which haploid maternal and paternal genomes carry different epigenetic marks, resulting in monoallelic transcription of a subset of genes which are expressed exclusively from either the maternal or paternal allele [1]. To date, over eighty imprinted genes have been identified in humans and mice [2]. Many of these genes are known to have critical roles in embryonic development and placentation [3] and have also been implicated in the regulation of postnatal behaviour [4-6]. The imprinted region on distal mouse chromosome 7 (Chr 7) shares syntenic homology with human chromosome 11p15.5, a region associated with Beckwith-Wiedemann syndrome (BWS) and Wilms tumor. BWS is an imprinted disorder that is commonly characterized by macroglossia, organomegaly, abdominal wall defects, unusual facial features, hemihypertrophy, hypoglycemia, exomphalos, ear and renal anomalies [7]. Roughly 5-10% of BWS patients are predisposed to a variety of childhood tumours, including hepatoblastoma, rhabdomyosarcoma, neuroblastoma, adrenal carcinoma, with Wilms tumor occurring most frequently [7,8].

Distal mouse Chr7 contains several key imprinted genes required for fetal development, two of which are also implicated in the etiology of BWS, the paternally expressed insulin-like growth factor-2 (*Igf2*) gene regulated by imprinting centre 1 (IC1, also known as H19 DMR) located upstream of the maternally expressed *H19 *gene and the maternally expressed cyclin-dependent kinase inhibitor 1C (*Cdkn1c*) which is regulated by imprinting centre 2 (IC2, also known as KvDMR1) located within intron 10 of the *Kcnq1 *gene [9,10]. In addition to *Cdkn1c*, the IC2 cluster contains at least eight other maternally expressed genes (*Ascl2*, *Cd81*, *Tssc4, Kcnq1, Slc22a18, Phlda2, Osbpl5*, and *Dhcr7*) [11-19]. Of these, *Ascl2*, *Cd81*, and *Osbpl5 *have been shown to be expressed and exclusively imprinted in the placenta [20,21]. The precise functions, if any, of *Cd81*, *Osbpl5*, *Tssc4*, *Kcnq1 *and *Slc22a18 *in the placenta remain to be elucidated. However, the remaining genes in the cluster (*Ascl2*, *Phlda2*, and *Cdkn1c*) have well-documented roles in placentation since knockouts of each of these genes result in drastic placental phenotypes. *Ascl2 *null mice fail to form a spongiotrophoblast layer, a defect leading to embryonic lethality at E10 [15,22]; *Phlda2 *null mice are viable but show an expanded spongiotrophoblast and placentomegaly [23]; *Cdkn1c *mutants possess varying phenotypes including a reduced labyrinth layer, expanded spongiotrophoblast, placentomegaly and perinatal lethality [24-26].

The mechanism of silencing of several genes both centromeric and distal to the position of the paternal IC2 over a large domain is not completely understood, though production and elongation of the ncRNA *Kcnq1ot1 *is thought to play a critical role in this process [27,28]. *Kcnq1ot1 *production is accompanied by recruitment of Polycomb group complexes [29] and acquisition of repressive histone modifications [20,21]. These observations suggest similarities with the action of other ncRNAs such as the regulation of *Igf2r *by the ncRNA *Airn *[30] and X-inactivation by *Xist *[31]. The deletion of the paternal IC2 leads to activation of IC2-regulated genes on the paternal chromosome and +/IC2KO mice possess a growth restriction phenotype at least in part because of biallelic expression of *Cdkn1c *[32,33]. The placentae of these animals were also reported to be smaller than their wild type litter mates [34]. Recent studies have also shown that IC2 contains CTCF-binding sites which are occupied only on the unmethylated paternal allele however it is still unclear what role these CTCF binding sites might play in silencing the paternal alleles of IC2-regulated genes [35]. It is also not known whether the methylated maternal IC2 allele is important for the activation of nearby genes.

We previously described an engineered truncation of distal Chr 7 with a breakpoint upstream of the *Ins2 *gene [36]. The resultant 2.7-Mb deletion, called DelTel7, removes the entire IC2-regulated domain along with the telomeric end of the Chr 7 domain, replacing it with an artificial telomere. When the DelTel7 allele is paternally inherited (+/DelTel7 hemizygotes), there is no obvious phenotype, showing absence of paternally expressed imprinted genes required for development or of a haploinsufficiency effect [36]. Maternal transmission of DelTel7 (DelTel7/+ hemizygotes) however, leads to a mid-gestational lethality, consistent with the deletion of all maternally expressed genes in the IC2-regulated sub-domain, and is also accompanied by abnormal placentation including the lack of spongiotrophoblast and an expanded trophoblast giant cell layer [36]. Despite a difference in phenotypic outcome, possibly attributable to the absence of imprinting of essential genes such as *ASCL2 *in humans [37,38], maternal inheritance of DelTel7 provides a mechanistic model for BWS, which is often associated with loss of expression of maternally expressed genes [9]. Remarkably, we also demonstrated that the lethality observed upon maternal inheritance of this truncation can be rescued in *trans *by a paternally inherited IC2KO allele, which was previously shown to abolish *Kcnq1ot1 *transcription and its associated epigenetic silencing [33]. The compound heterozygous DelTel7/IC2KO pups survive to term but it is unclear whether any placental abnormalities or defects in placental imprinted gene expression remain in the double mutants. Because of the importance of several IC2-regulated genes in placental function and development, we present here a characterization of the rescued DelTel7/IC2KO placentae to determine whether they are comparable to their wild type litter mates and whether deletion of IC2 leads to a full rescue of the placental phenotype caused by the loss of maternal genes from the DelTel7 allele. Our assessment would have direct implications for BWS patients, since placental abnormalities usually accompany BWS fetuses [39-41]. By examining the architecture, vasculature, and imprinted gene expression of the rescued DelTel7/IC2KO placentae we sought to determine whether it is possible to correct imprinting defects by restoring gene expression for a large number of genes in *trans *and thereby to further elucidate mechanisms of IC2-mediated epigenetic silencing.

## Results

### Loss of imprinted gene expression in DelTel7/+ placentae

**Figure 1:**
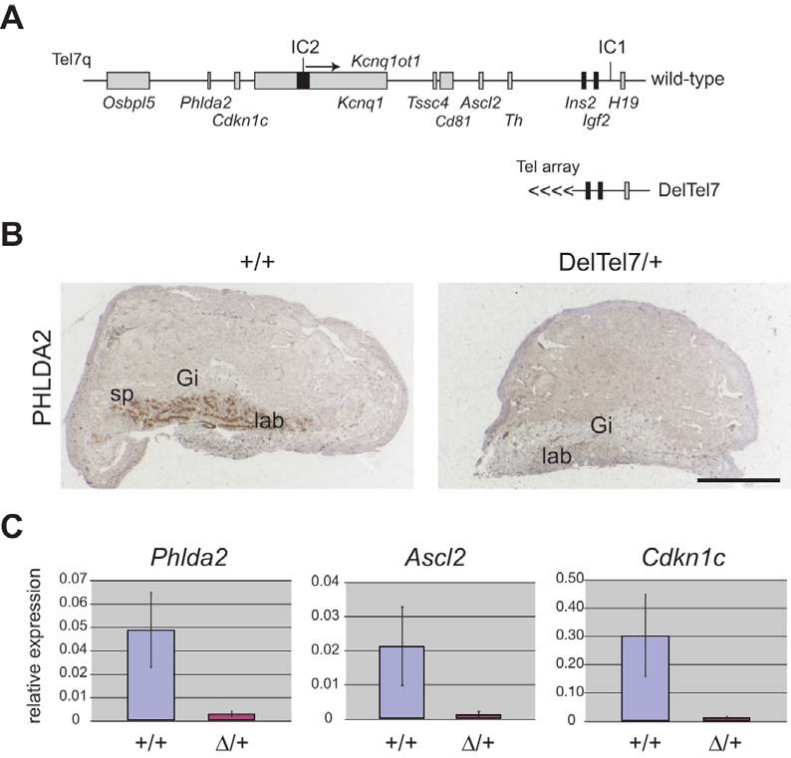
**Expression of IC2-regulated genes in DelTel7/+ at E9.5**. A) Diagram of distal mouse chromosome 7 (Chr 7) region showing the locations of the imprinting centres 1 (IC1) next to *H19 *and 2 (IC2) in intron 10 of the *Kcnq1 *gene, as well as the protein-coding genes known to be regulated by IC2 (*Osbpl5, Phlda2, Cdkn1c, Kcnq1, Tssc4, Cd81*, and *Ascl2*). This entire IC2-regulated cluster is deleted in the DelTel7 allele in which an array of telomere repeats (Tel array) was introduced distal of *Ins2 *[36]. B) PHLDA2 immunohistochemistry (IHC) on sections of wild type (+/+) and maternal hemizygous (DelTel7/+) placentae at E9.5. PHLDA2 IHC was performed on two placentae of each genotype. The PHLDA2 protein, localized to the labyrinth of the wild type placenta, is absent in DelTel7/+ mutants. Note the lack of spongiotrophoblast and expanded giant cell layer in the DelTel7/+ placenta. Scale bar: 1 mm. sp: spongiotrophoblast, lab: labyrinth, Gi: giant cells. C) qRT-PCR analysis of the imprinted genes *Phlda2*, *Ascl2*, and *Cdkn1c*, in E9.5 DelTel7/+ placentae reveals virtually no expression from the intact paternal allele. Placentae were assessed at E9.5 before the embryonic lethality of the DelTel7/+ observed at around E10.5. qRT-PCR was performed on five DelTel7/+ placentae and five wild type placentae with three technical replicates per individual placentae. Expression is relative to the reference housekeeping gene, *Peptidylprolyl isomerase A *(*Ppia*). The error bar is the standard deviation from five biological replicates, assayed in triplicates. p < 0.02 for each qRT-PCR (*t*-test, unpaired, two-tailed).

The IC2 imprinted domain of distal Chr 7 contains at least several maternally expressed mRNA genes and spans almost 800 kb. The entire domain is deleted in the DelTel7 allele, a truncation of Chr7 with a breakpoint upstream of *Ins2 *and deleting the last 2.7 Mb of Chr7 (Figure 1A). We previously showed that maternal transmission of DelTel7 leads to abnormal placentation and embryonic lethality at E10.5, whereas paternal hemizygotes are viable [36]. We show here by immunohistochemistry for PHLDA2 (Figure 1B) as well as quantitative RT-PCR (qRT-PCR) for the imprinted genes *Phlda2, Ascl2*, and *Cdkn1c*, that at E9.5 DelTel7/+ maternal hemizygous placentae are deficient for expression of these IC2-regulated genes. Placentae were assessed at E9.5, before the embryonic lethality of the DelTel7/+ embryos. We found only basal transcription levels of expression by qRT-PCR for these IC2-regulated genes in DelTel7/+ placentae (Figure 1C). Wild type levels were quite varied between samples, suggesting dynamic changes in expression at this early developmental stage or effects the genetic heterogeneity present in these crosses on mixed background. Note the lack of spongiotrophoblast and the expanded giant cell layer in the DelTel7/+ placenta (Figure 1B), consistent with what has been published previously regarding the DelTel7/+ phenotype [36].

Other then the entire IC2 sub-domain, DelTel7 also deletes the very distal end of Chr7. This region contains close to 20 annotated genes. Unlike genes in the IC1 and IC2 domains, we find that most of these distal genes are not expressed in the placenta at significant levels (Additional file 1). The absence of an obvious phenotype in paternal hemizygotes for DelTel7 argues against haploinsufficiency effects in these mutants.

### Germline transmission from DelTel7/IC2KO mice

**Figure 2:**
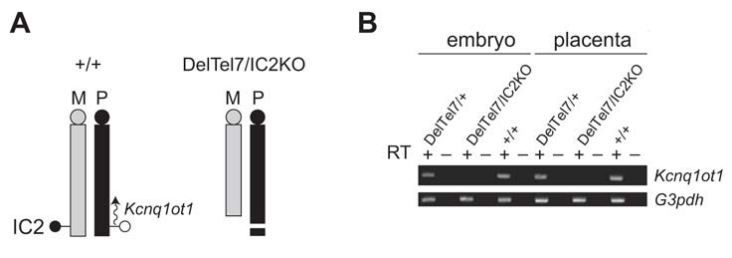
**Structure of the DelTel7 allele and absence of *Kcnq1ot1 *in rescued DelTel7/IC2KO embryos**. A) Simplified representation of the structure of distal Chr7 in the DelTel7/IC2KO rescued animals, revealing the DelTel7 truncation on the maternal allele and the absence of IC2 and lack of activation of *Kcnq1ot1 *expression on the paternal allele. M = maternal; P = paternal. C) RT-PCR demonstrating lack of *Kcnq1ot1 *expression in E9.5 DelTel7/IC2KO embryo and placenta.

IC2 is comprised of CpG-rich sequences within intron 10 of the *Kcnq1 *gene and functions in gene silencing, at least in part, by serving as a promoter for the antisense ncRNA *Kcnq1ot1*. We previously showed that paternal transmission of the IC2KO allele can rescue the embryonic lethality phenotype of DelTel7/+ embryos [36]. The rescued DelTel7/IC2KO mice are missing the genes that are normally maternally expressed (they are deleted in DelTel7) but expression of these genes is now supplied from the paternal allele, since the IC2KO allele no longer produces *Kcnq1ot1 *(Figure 2a) [33]. We confirmed the absence of *Kcnq1ot1 *expression in DelTel7/IC2KO embryos and placentae by strand-specific RT-PCR (Figure 2B). Phenotypic analysis of these compound heterozygous mice therefore allows us to ask the following questions: (*i*) Are all the maternally-expressed genes required for normal placental development expressed from the IC2KO paternal allele? (*ii*) Is the methylated IC2 required in the regulation of maternally-expressed genes on the maternal allele in wild type embryos?

We have previously shown that DelTel7/IC2KO pups survive to term, are recovered at the expected frequency and are indistinguishable from their wild type litter mates at birth (Oh et al, 2008). We now report that DelTel7/IC2KO males and females are fertile and pass on the mutant alleles at expected frequencies in their offspring. DelTel7/IC2KO males crossed with wild type (+/+) females produce litters of +/DelTel7 and +/IC2KO pups at roughly equal frequencies (Table 1). DelTel7/IC2KO females crossed with wild type males produce litters of only the IC2KO/+ genotype as expected, since the DelTel7/+ genotype is embryonic lethal (Table 1). Mice produced from these crosses do not possess any obvious developmental abnormalities and are able to survive to adulthood. These results demonstrate that the maintenance of imprinting is faithfully recapitulated in each generation and that there are no aberrant effects of transmitting either allele in the context of the compound heterozygotes.

**Table 1:** Transmission of the DelTel7 (Δ) and IC2KO alleles from compound heterozygotes

male	female	progeny	
		
		Δ het	IC2KO het
+/+	Δ/IC2KO	0	12
Δ/IC2KO	+/+	40	37

### DelTel7/IC2KO placentae exhibit normal architecture

**Figure 3:**
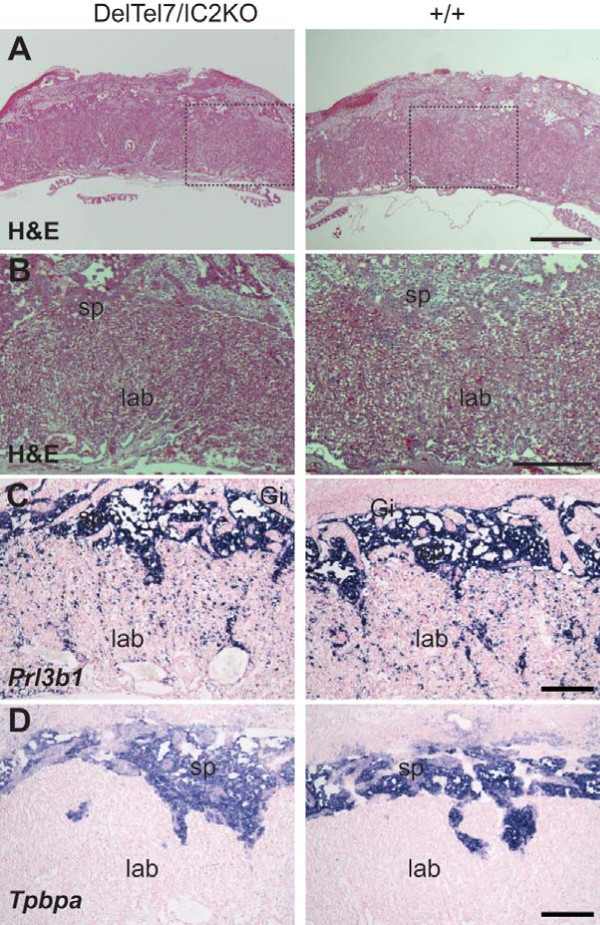
**Overall placental architecture of the DelTel7/IC2KO placentae at E14.5 by representative H&E and ISH**. A) H&E stain of DelTel7/IC2KO placentae (n = 5) compared with wild type (+/+; n = 5). Scale bar: 1 mm. B) Zoom-in of boxed regions in panel A. Scale bar: 0.5 mm. C) Analysis of *Prl3b1 *(*Pl-II*) expression by ISH of DelTel7/IC2KO placentae (n = 5) compared with wild type (n = 6). Scale bar: 0.25 mm. D) *Tpbpa *(4311) ISH of DelTel7/IC2KO placentae (n = 4) compared with wild type (n = 4) Scale bar: 0.25 mm.

Because the DelTel7/+ genotype results in abnormal placentation, we investigated the histology of several DelTel7/IC2KO placentae compared to wild type litter mates to determine whether there were any abnormalities in placentation in the compound heterozygotes. Hematoxylin and eosin stain (H&E) revealed no apparent abnormalities between DelTel7/IC2KO and wild type placentae at E14.5, suggesting that the DelTel7/IC2KO placentae displayed normal placental architecture (Figure 3A and 2B). To document this in greater detail, we used in situ hybridization (ISH) and immunohistochemistry (IHC) to analyze the distribution of the several lineage markers in wild type and DelTel7/IC2KO placentae. Neither ISH for the placental lactogen-II gene *Prl3b1 *(*Pl-II*), a mid- to late gestation marker of both the giant cell layer and mononucleated trophoblast cells of the labyrinth (Figure 3C) [42], nor ISH for *Tpbpa *(4311), a marker of the spongiotrophoblast layer (Figure 3D) [43] revealed any overall differences in the these layers between the two genotypes.

**Figure 4:**
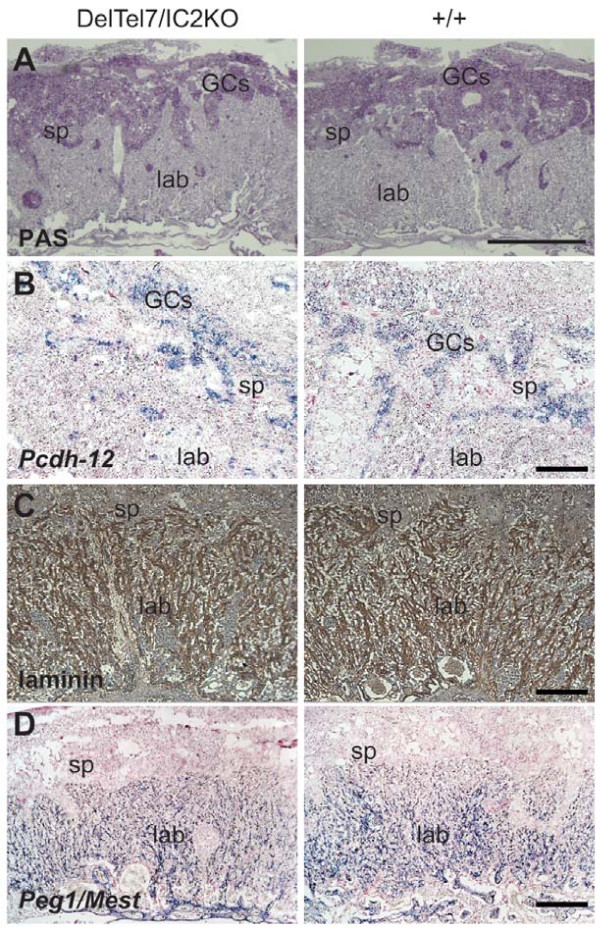
**Placental glycogen cells and labyrinthine architecture of the DelTel7/IC2KO placentae at E14.5**. A) Trophoblast glycogen cells are highlighted by periodic acid Schiff (PAS) staining of DelTel7/IC2KO placentae (n = 4) compared with wild type (n = 4). Scale bar: 1 mm. B) Detection of *Pcdh12*, a glycogen cell marker, by ISH on DelTel7/IC2KO placentae (n = 4) compared with wild type (n = 4). Scale bar = 0.25 mm. C) Extraembryonic mesoderm of the labyrinth layer detected by laminin IHC on DelTel7/IC2KO placentae (n = 4) compared with wild type (n = 4). Scale bar = 0.25 mm. D) *Peg1/Mest *ISH of DelTel7/IC2KO placentae (n = 4) compared with wild type (n = 4). Scale bar = 0.25 mm. GCs: glycogen cells; sp: spongiotrophoblast; lab: labyrinth.

To determine whether there was a defect in the generation of the glycogen cell (GC) lineage in the DelTel7/IC2KO placentae, both Periodic acid Schiff (PAS) stain and ISH for *Protocadherin-12 *(*Pcdh12*), a gene specifically expressed in GCs [44] were performed (Figure 4A and 4B). These analyses revealed no difference in the overall number or distribution of GCs between the two genotypes. For a detailed examination of the labyrinth, we performed IHC for laminin, a marker of the basement membrane in the labyrinth used for identifying fetal blood vessels (Figure 4C) [45] and ISH for the imprinted paternally expressed gene 1 (*Peg1/Mest*) which is also expressed in the fetal endothelial cells that line the fetal capillaries (Figure 4D). No abnormalities were observed in the cell lineages analyzed or in the fetal capillaries of the labyrinth of DelTel7/IC2KO placentae. Our results show that DelTel7/IC2KO placentae are phenotypically similar to wild type litter mates for the various representative markers analyzed.

**Figure 5:**
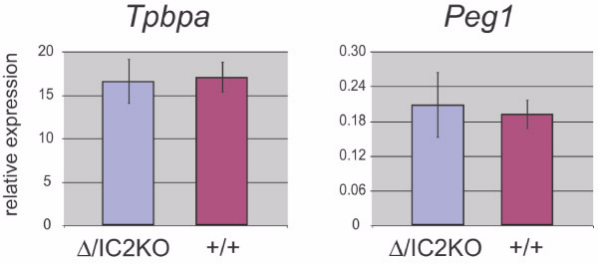
**Expression of placental marker in E14.5 placentae**. qRT-PCR analysis of the expression levels of the placental marker *Tpbpa *(4311) and *Peg1 *in wild type (+/+) and rescued DelTel7/IC2KO (Δ/IC2KO) placentae at E14.5. Expression is relative to the reference housekeeping gene, *Peptidylprolyl isomerase A *(*Ppia*). The error bar is the standard deviation from three biological replicates, assayed in triplicates. p > 0.6 for each qRT-PCR (*t*-test, unpaired, two-tailed).

To confirm the results of these marker analyses using an independent method, we also performed expression analysis for *Tpbpa *(4311) and *Peg1 *qRT-PCR and observed no difference between the two genotypes (Figure 5).

### DelTel7/IC2KO placentae exhibit normal vasculature

**Figure 6:**
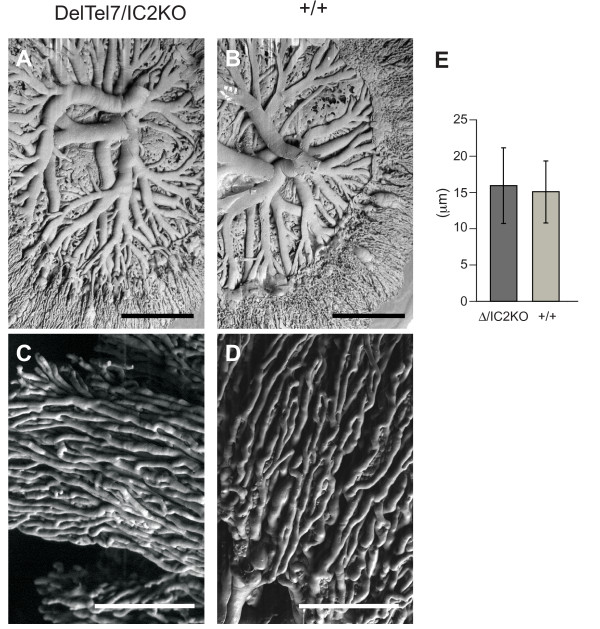
**Feto-placental vasculature of DelTel7/IC2KO placentae at E14.5**. A) Representative scanning electron micrographs of DelTel7/IC2KO fetal vasculature casts of whole placentae (n = 4) compared with B) wild type litter mates (n = 4). C) In-depth view of fetal capillaries of DelTel7/IC2KO compared with D) wild type. E) Morphometric analysis of fetal capillary diameters between DelTel7/IC2KO (Δ/IC2KO; 226 measurements from 4 placentae) and wild type (+/+; 144 measurements from 4 placentae) placentae. Scale bar for A) and B) = 1 mm. Scale bar for C) and D) = 250 microns. Note that in B) the umbilical vein has been dissected away at the primary branch points.

Because we had no indication from our lineage marker analysis that the DelTel7/IC2KO placentae had a drastically abnormal phenotype, we explored the possibility that they may possess a subtle placental phenotype. One of the most common subtle placental phenotypes is an undervascularized labyrinth, otherwise known as a "small" labyrinth [46]. Thus in addition to examining placental architecture, we also assessed whether the vascularization of the DelTel7/IC2KO placentae was perturbed using the vascular corrosion casting method [47]. We found no apparent differences in the overall structure and number of capillaries in the fetal vasculature of DelTel7/IC2KO placentae compared to wild type litter mates at E14.5 (Figure 6A-D). Moreover, morphometric analysis of the average capillary diameter revealed no overall difference between DelTel7/IC2KO placentae and wild type placentae (Figure 6E), with both having an average of approximately 15 microns. This measure is in agreement with the previously documented average capillary diameter in wild type placentae [48].

### DelTel7/IC2KO placentae exhibit proper expression of imprinted genes

**Figure 7:**
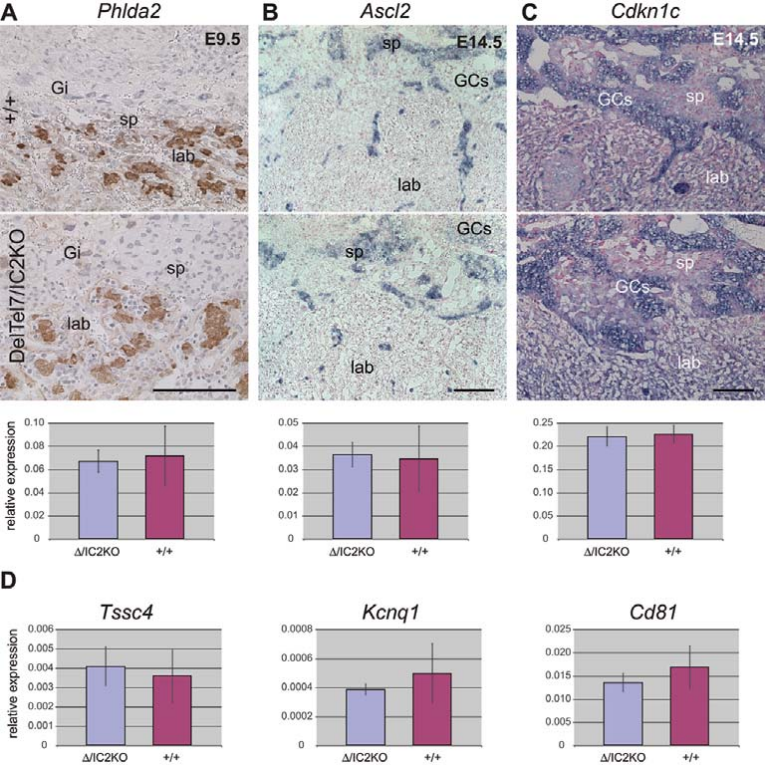
**Imprinted gene expression in DelTel7/IC2KO placentae**. The expression of three IC2-regulated genes was analyzed in wild type (+/+) and rescued (DelTel7/IC2KO) placentae on placental section (top) and by qRT-PCR on total placental RNA (bottom). A) *Phlda2 *expression analyzed by IHC on paraffin sections of wild type (n = 4) and mutant (n = 4) placentae at E9.5. The qRT-PCR was performed on E9.5 placental RNA; DelTel7/IC2KO and +/+. B) *Ascl2 *expression analyzed by ISH on frozen placental sections at E14.5; n = 4 for DelTel7/IC2KO and wild type. *Ascl2 *expression by qRT-PCR at E14.5 on mutant placentae compared with +/+. C) *Cdkn1c *expression in DelTel7IC2KO placentae (n = 9) at E14.5 compared with +/+ (n = 9). *Cdkn1c *expression by qRT-PCR at E14.5 on mutant placentae compared with +/+. Sense probes not shown. GCs = glycogen cells; lab = labyrinth; sp = spongiotrophoblast; Gi = trophoblast giant cells. The blue and brown stains show gene and protein expression, respectively. All scale bars: 0.25 mm. All qRT-PCRs at E9.5 were performed on five DelTel7/IC2KO placentae and five wild type placentae with three technical replicates per individual placentae. All qRT-PCRs at E14.5 were performed on three DelTel7/IC2KO placentae and three wild-type placentae with three technical replicates per individual placentae. All expression results by qRT-PCR are shown normalized to the *Ppia *reaction and results are shown ± SD. First, the average from each placenta was calculated from technical replicates from the same cDNA. Then the SD was calculated from the average of biological replicates per genotype. For the three genes tested the differences between wild type and mutant placentae are not statistically significant (p > 0.7). D) qRT-PCR of the other imprinted genes in the IC2 domain, *Tssc4*, *Kcnq1 *and *Cd81*, in E9.5 DelTel7/IC2KO placentae reveals no overall difference from wild type (p > 0.2). qRT-PCR was performed as described above on five placentae of each genotype.

To determine whether DelTel7/IC2KO placentae express the IC2-regulated genes from the paternal allele in a normal cell-type specific manner, we analyzed the expression of three key genes in the distal Chr 7 region strongly associated with placentation: *Phlda2*, *Ascl2*, and *Cdkn1c *(Figure 7). We chose to look at stages in which the expression level of each of the genes is high or at its highest according to our own observations as well as previously published work [23,49,50]. IHC for *Phlda2 *on E10.5 DelTel7/IC2KO placentae revealed exclusive expression in trophoblast cells of the labyrinth, consistent with wild type litter mates. ISH for *Ascl2 *on DelTel7/IC2KO placentae revealed exclusive expression in cells of the spongiotrophoblast layer with some expression in glycogen cells (GCs), consistent with wild type litter mates. ISH for *Cdkn1c *on DelTel7/IC2KO placentae revealed high expression in GCs and low expression throughout other cell types in the labyrinth, as seen in wild type litter mates (Figure 7). The relative expression level of each of these genes was confirmed by quantitative RT-PCR (qRT-PCR). No statistically significant differences in expression levels between DelTel7/IC2KO and wild type placentae were observed for *Phlda2*, *Ascl2 *and *Cdkn1c *at the stages analyzed (Figure 7A-C). Additionally, an examination of expression level of several of the other imprinted genes in the IC2 region (*Tssc4*, *Kcnq1*, and *Cd81*) by qRT-PCR at E9.5 revealed no significant differences between DelTel7/IC2KO and wild type placentae (Figure 7D).

## Discussion

Placental abnormalities such as placentomegaly [41] and placental mesenchymal dysplasia [40] are commonly observed in mothers carrying BWS fetuses. Mouse models of the BWS imprinted region such as the *Igf2 *transgenic mouse model [51] and *Cdkn1c *null mouse models [24-26] which accurately recapitulate several phenotypic characteristics of the human disorder, are also accompanied by severe placental abnormalities, highlighting the importance of determining whether any placental abnormalities exist in the DelTel7/IC2KO mouse model. In these compound heterozygotes, IC2-regulated imprinted genes such as *Cdkn1c *are deleted from the maternal allele and can only be expressed from the paternal chromosome because of the IC2 deletion. Our studies have shown that rescuing the deleted IC2 imprinted domain results in appropriate placentation with respect to placental architecture, placental vasculature and imprinted gene expression. Our findings lead us to propose that rescuing an imprinting defect can also rescue the associated placental phenotype, which may have implications for BWS patients who have associated placental defects.

It has been postulated that mechanisms other than transcription and elongation of *Kcnq1ot1 *may influence repression of the paternal distal Chr 7 genes. For example, *Cdkn1c *imprinting has previously been shown to be controlled by an element some distance away from the gene itself in a bacterial artificial chromosome (BAC) mouse model of *Cdkn1c*, which failed to reproduce appropriate *Cdkn1c *imprinting [49]. In another study, a yeast artifical chromosome (YAC) transgene containing the entire IC2 domain was shown to recapitulate faithful imprinting of most of the IC2-regulated genes (*Phlda2*, *Slc22a18, Kcnq1 *and *Cdkn1c*), but loss of imprinting of *Ascl2 *and *Tssc4 *was observed when the YAC was paternally inherited [52]. Both of these genes lie centromeric to IC2 and suggests that perhaps these genes require additional control elements beyond *Kcnq1ot1 *transcription on the paternal allele that are absent on the YAC transgene. The results of our study differ from the above in that we have demonstrated that the absence of *Kcnq1ot1 *transcription and/or deletion of other sequences in IC2 not only leads to full and normal expression of the paternal distal Chr 7 genes but to a full rescue of our DelTel7 placental phenotype, suggesting that this locus is the single most critical component in maintaining epigenetic silencing in this region. Since both normal placental phenotype and placental expression of IC2 genes are observed in DelTel7/IC2KO conceptuses, our results also suggest that the normally methylated maternal allele of IC2 and its potential association with methyl-CpG-binding factors is not necessary in regulating the expression of the genes analyzed.

The placentomegaly resulting from maternal inheritance of a *Phlda2 *null allele has been shown to be almost completely rescued by paternal inheritance of the same IC2KO allele used in this study [34], providing evidence of proper *Phlda2 *expression levels from the paternal IC2KO allele. To the best of our knowledge, the DelTel7/IC2KO model is the first example of rescue of a large disrupted imprinted region, not just a single gene. We demonstrate that restoration of gene expression for a large number of genes in *trans *is possible and our work demonstrates the potential ability to rectify placental abnormalities caused by imprinting defects. Future studies include developing RNAi to knock down *Kcnq1ot1 *during preimplantation stages in DelTel7 pregnant females to determine if this can rescue placental and embryonic phenotypes. Such experiments would suggest that it may be possible to therapeutically target the ncRNA as a step towards aiding BWS patients with maternal defects at IC2 that result in maternal *Kcnq1ot1 *reactivation.

## Conclusions

The DelTel7 deletion offers an opportunity to assess the phenotypic consequences of loss of function for a cluster of maternally expressed genes on distal Chr7. Here we show that deletion of the *Kcnq1ot1 *promoter and nearby sequences (IC2KO) on the paternal allele can fully rescue the placental phenotype associated with maternal inheritance of DelTel7. In the viable conceptuses, monoallelic expression of imprinted genes is reversed and is now provided by the non-silencing alleles on the paternal IC2KO chromosome. Our results show that all the genes required for normal placentation, missing in the DelTel7 allele are regulated by IC2 and the ncRNA *Kcnq1ot1 *on the paternal allele during normal development. The methylated maternal allele of IC2 is not implicated in the regulation of these genes. Our work establishes the possibility of rescuing imprinted disorders such as BWS in *trans*, by interfering with the expression or the long-range silencing function of the regulatory ncRNA *Kcnq1ot1*.

## Methods

### Mice and genotyping

For all genotypes, the maternal allele is always given first. We previously presented the generation of the DelTel7 mouse line and genotyping of the DelTel7 allele [36]. Genotyping of the IC2KO allele (*KvDMR1*) is as previously described [33]. Females heterozygous for the deletion allele (+/DelTel7) maintained on the outbred CD-1 background were mated to +/IC2KO males on the C57BL/6 background and placentae at various developmental stages were dissected. All animal experiments were performed under certificate A07-0160 from the UBC Animal Care Committee and complied with the national CCAC guidelines to the ethical care and use of experimental animals.

### RT-PCR and quantitative PCR

E9.5 and E14.5 placentae were dissected in cold PBS and snap frozen on dry ice. Placental RNA was extracted by Trizol (Invitrogen) and cDNA was synthesized using SuperScript II (Invitrogen). For *Kcnq1ot1 *detection, a gene specific primer for *Kcnq1ot1 *was designed (*Kcnq1ot1 *GSP 5'CACATACACACACCCAACTCG 3') and PCR was performed under standard conditions. Primers for the *Ppia *reaction have been previously described [53]. Quantitative RT-PCR was performed in technical triplicate on five individual placental samples of each genotype at E9.5 and three samples at E14.5 (+/+ and DelTel7/IC2KO) for all genes analyzed. For *Cdkn1c*, forward primer (5' GCGCAAACGTCTGAGATGAG 3') and reverse primer (5' CAGCCGAAGCCCAGAGTTC 3') were used. For *Phlda2*, forward primer (5' CCCGCCAAGGAGCTGTTT 3') and reverse primer (5' CCTTGTAATAGTTGGTGACGATGGT 3') were used. For *Ascl2*, forward primer (5' TCCTGGTGGACCTACCTGCTT 3') and reverse primer (5' AGGTCAGTCAGCACTTGGCATT 3') were used. For *Tssc4*, forward primer (5' ACGGGTGTCAGGTCGTATGG 3') and reverse primer (5' TGAGGGAGACGGTGTCAGAAG 3') were used. For *Kcnq1*, forward primer (5' AGAAGCAGAGGCAGAAGCACT 3') and the reverse primer has been previously described [11]. For *Cd81*, forward primer (5' CTGGCTGGAGGCGTGATC 3') and reverse primer (5' TGGGTGCCGGTTTGTTTC 3') were used. For *4311*, forward primer (5' CAGCTTTGGACATCACAGGTACTT 3') and reverse primer (5' TGCGCTTCAGGGACTATAGCA 3') were used. For *Peg1*, forward primer (5' TGTCCATCCCCATTCATTTT 3') and reverse primer (5' GAGTTCCAGCTGCCTGATTC 3') were used.

### Immunohistochemistry

E9.5 and E14.5 placentae were dissected in cold PBS and fixed in fresh 4% paraformaldehyde overnight at 4°C. Paraffin-embedded sections were baked, cleared of paraffin and rehydrated by a series of xylene and ethanol washes. Antigen retrieval was done by microwaving slides in 1 mM EDTA, pH 7.5. Endogenous peroxidase blocking was done in 0.3% hydrogen peroxidase for 30 minutes. Blocking was done with 5% goat normal serum (Vector Labs) in 1× TBS-T with 0.5% BSA for 30 minutes. To detect *Phlda2 *protein in the labyrinth layer, and to identify fetal capillaries of the labyrinth layer, a 1:1000 dilution of the *Phlda2 *antibody and a 1:25 dilution of rabbit polyclonal anti-laminin antibody (Sigma) were used, respectively. Incubation was done overnight at room temperature for *Phlda2 *antibody and 1 hour for laminin antibody. A 1:500 dilution of the biotinylated anti-rabbit IgG secondary antibody (Vector Labs) was used for 1 hour and after a series of TBS-T washes, the staining was performed with the Vectastain Elite ABC kit and DAB substrate (Vector Labs). Counterstain was done with hematoxylin (Sigma) for about 1 minute and the slides were dehydrated in a series of ethanol and xylene washes before mounting with Entellan mounting medium (EM Science) and coverslipped with glass.

### In situ hybridization

Antisense and sense strand probes were DIG-labelled using 10× DIG labeling mix (Roche) and T7, SP6, or T3 RNA polymerases (Roche). Probes were digested with DNaseI and purified by LiCl precipitation. Placentae at various stages were dissected in PBS and fixed in fresh 4% paraformaldehyde/1× PBS (RNase free) overnight at 4°C. The next day, they were washed in PBS and left overnight in 30% sucrose in PBS. They were allowed to equilibrate in OCT (Tissue-Tek) for half an hour at room temperature before embedding in OCT on dry ice. The blocks were stored at -80°C until ready to be used. Cryostat sections were cut at 10 microns and stored at -20°C until next day when hybridized. Sections were first thawed at 50°C, fixed in 4% PFA, treated with Proteinase K (final concentration of 40 ug/ml) and acetylated in acetic anhydride to reduce background. Sections were then prehybridized in hybridization buffer (50% formamide, 5× SSC, 5× Denhardt's, 0.25 mg/ml Yeast tRNA) at 60°C for 3 hours before hybridization at 55°C overnight. The next day, non-specific probe was removed by a series of SSC washes and treated with RNase A before blocking for 1 hour in 1% blocking buffer (Roche) at 37°C. Sheep anti-DIG-alkaline phosphatase conjugated antibody (1:2000) (Roche). The BCIP (Roche) and NBT (Roche) reaction was used to detect the signal. After three 1× PBS washes, sections were fixed in a solution containing 3.7% formaldehyde, counterstained with nuclear fast red (Sigma) and dehydrated before mounting with Entellan mounting medium and coverslipping with glass. Multiple sections (between 16 and 36) were analyzed per genotype per marker.

### Placental fetal vasculature casts

Fetal vascular corrosion casts of E14.5 placentae were generated by following the procedure previously described by Whiteley *et al*. [47]. Vascular casts were analyzed by SEM and morphometric analysis of capillary diameter was carried out using Openlab software (Improvision).

## List of abbreviations used

BWS: Beckwith-Wiedemann syndrome; CCAC: Canadian Council on Animal Care; Chr 7: mouse chromosome 7; GC: trophoblast glycogen cells; IC2: imprinting center 2, KvDMR1; IHC: immunohistochemistry; ISH: *in situ *hybridization; Mb: megabases; PAS: periodic acid Schiff; RT-PCR: reverse transcription polymerase chain reaction; SEM: scanning electron microscope; YAC: yeast artificial chromosome.

## Authors' contributions

ROM performed the experiments and drafted the manuscript. AB performed the placental corrosion cast experiments. KYL performed and analyzed the microarray analysis. MJH provided the KvDMR1 KO (IC2KO) mice and critically revised the manuscript. LL conceived the study, helped with experimental design and helped drafting the manuscript. All authors read and approved the final manuscript.

## Supplementary Material

Additional file 1 **Expression of genes on distal mouse chromosome 7 in the placenta**. Four placental samples were collected from E15.5 male conceptuses from C57Bl/6 mice. RNA was extracted from whole placenta and sent for microarray analysis at the McGill University and Genome Quebec Innovation Centre. Expression data was obtained from Illumina MouseRef-8 v2.0 Beadchip and pre-processed by the lumi normalization within the FlexArray software [refs. [1,2]]. IC1: Imprinting centre 1. IC2: Imprinting centre 2. *Ppia *and *Gapdh *are housekeeping genes. *Xist *and *Olfr541 *are not expressed in male mouse placenta. *Olfr541 *is located on chromosome 7, ~2 Mb upstream of *Igf2*. **1**. lumi: a pipeline for processing Illumina microarray. Du P, Kibbe WA, Lin SM. *Bioinformatics*. 2008 Jul 1; **24**(13):1547-8. **2**. Model-based variance-stabilizing transformation for Illumina microarray data. Lin SM, Du P, Huber W, Kibbe WA. *Nucleic Acids Res*. 2008 Feb; **36**(2):e11 Click here for file
