# Supplementary figures and images for: Rescue of placental phenotype in a mechanistic model of Beckwith-Wiedemann syndrome
Source: BMC Dev Biol. 2010 May 11;10:50. doi: 10.1186/1471-213X-10-50 (PMC2881899; doi:10.1186/1471-213X-10-50)

# relative expression units

7.8 9.8 11.8 13.8 15.8

IC1

IC2

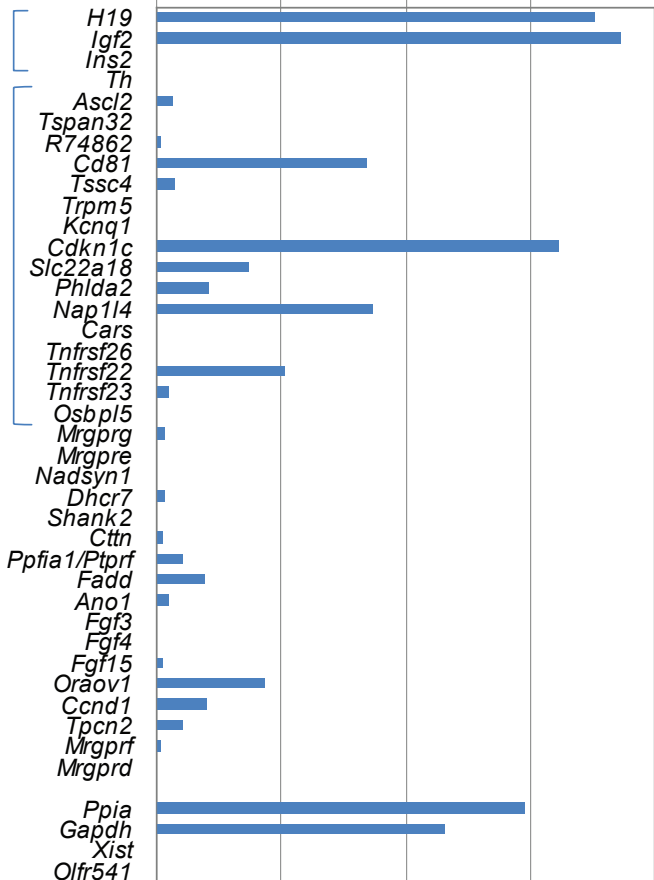

Supplement: Additional file 1 — Expression of genes on distal mouse chromosome 7 in the placenta. Four placental samples were collected from E15.5 male conceptuses from C57Bl/6 mice. RNA was extracted from whole placenta and sent for microarray analysis at the McGill University and Genome Quebec Innovation Centre. Expression data was obtained from Illumina MouseRef-8 v2.0 Beadchip and pre-processed by the lumi normalization within the FlexArray software [refs. [1,2]]. IC1: Imprinting centre 1. IC2: Imprinting centre 2. Ppia and Gapdh are housekeeping genes. Xist and Olfr541 are not expressed in male mouse placenta. Olfr541 is located on chromosome 7, ~2 Mb upstream of Igf2. 1. lumi: a pipeline for processing Illumina microarray. Du P, Kibbe WA, Lin SM. Bioinformatics. 2008 Jul 1; 24(13):1547-8. 2. Model-based variance-stabilizing transformation for Illumina microarray data. Lin SM, Du P, Huber W, Kibbe WA. Nucleic Acids Res. 2008 Feb; 36(2):e11 [file 1471-213X-10-50-S1.PDF]
